# Effect of Docosahexaenoic Acid Encapsulation with Whey Proteins on Rat Growth and Tissue Endocannabinoid Profile

**DOI:** 10.3390/nu15214622

**Published:** 2023-10-31

**Authors:** Jun Wang, Jordane Ossemond, Yann Le Gouar, Françoise Boissel, Didier Dupont, Frédérique Pédrono

**Affiliations:** National Research Institute for Agriculture, Food and Environment (INRAE), L’Institut Agro Rennes-Angers, Science and Technology of Milk and Egg (STLO), 35042 Rennes, France; jun.wang@inrae.fr (J.W.); jordane.ossemond@inrae.fr (J.O.); yann.le-gouar@inrae.fr (Y.L.G.); francoise.boissel@institut-agro.fr (F.B.); didier.dupont@inrae.fr (D.D.)

**Keywords:** docosahexaenoic acid (DHA), encapsulation, endocannabinoid, *N*-acyl ethanolamide, fasting, brain, heart, rat

## Abstract

Modifying the food structure allows a nutrient to be delivered differently, which can modify not only its digestion process but also its subsequent metabolism. In this study, rats received 3 g of omelette daily containing docosahexaenoic acid (DHA) as crude oil or previously encapsulated with whey proteins, whereas a control group received a DHA-free omelette. The results showed that DHA encapsulation markedly induced a different feeding behaviour so animals ate more and grew faster. Then, after four weeks, endocannabinoids and other *N*-acyl ethanolamides were quantified in plasma, brain, and heart. DHA supplementation strongly reduced endocannabinoid derivatives from omega-6 fatty acids. However, DHA encapsulation had no particular effect, other than a great increase in the content of DHA-derived docosahexaenoyl ethanolamide in the heart. While DHA supplementation has indeed shown an effect on cannabinoid profiles, its physiological effect appears to be mediated more through more efficient digestion of DHA oil droplets in the case of DHA encapsulation. Thus, the greater release of DHA and other dietary cannabinoids present may have activated the cannabinoid system differently, possibly more locally along the gastrointestinal tract. However, further studies are needed to evaluate the synergy between DHA encapsulation, fasting, hormones regulating food intake, and animal growth.

## 1. Introduction

Docosahexaenoic acid (DHA) is an essential omega 3 fatty acid mainly known for its beneficial effects on brain [1,2] and heart [3,4] functions. It can be synthesised de novo from omega 3 precursors or be directly provided by food. Direct dietary intake is particularly preferred due to the low rate of conversion of omega 3 precursors into DHA. Thus, two DHA delivery strategies have been developed to optimise its physiological effects. The first is molecular delivery, which consists of offering DHA in a rather phospholipid form, to the detriment of DHA esterified in triglycerides or in the form of an ethyl ester [5,6,7]. The second is food delivery, which promotes the supply of DHA in the encapsulated form in a food matrix chosen for better bioavailability of DHA afterwards [8,9]. It is this second strategy that we have developed. In the present study, DHA oil has been encapsulated through Pickering emulsion made of heat-denatured whey proteins as described by Wang et al. in 2020 [10]. Other strategies have been shown to be efficient for encapsulating DHA. Micelles made of chymotrypsin-hydrolysed dietary proteins (ovalbumin, myosin, 7S soy globulin or β-lactoglobulin) provided spatial stability and improved solubility, and stability against thermal and ultraviolet light for DHA, improving the uptake by Caco-2 cells [11]. DHA has also been protected from oxidation by encapsulation in chitosan-tripolyphosphate nanoparticles [12], caseinate-alginate microparticles [13], monolayered (lecithin with maltodextrine) and multilayered (lecithin with chitosan-maltodextrine) microcapsules [14], or alginate beads [15], demonstrating that it is currently a hot topic. Thereby, whey protein-based microcapsules of DHA oil were incorporated into eggs and baked thereafter as omelettes. Rats were then fed daily for 4 weeks with these DHA-enriched omelettes, the DHA oil being encapsulated or not. In the first part, we have already shown that the encapsulation of DHA oil leads to a modification of the profile of tissue fatty acids and also of the oxylipins derived from these fatty acids [16]. Other in vitro results showed that as much DHA was released during digestion after one hour of lipase activity when the DHA oil was encapsulated as after two hours of digestion when the DHA oil was not encapsulated [17]. Therefore, the bioaccessibility of DHA was significantly improved by the encapsulation of DHA oil in omelettes. This effect was inherent to the enzymatic accessibility favoured by the smaller lipid droplet size in the case of DHA oil encapsulation. Consequently, this modification of digestion generates different metabolic effects of DHA depending on the form of food intake.

Alongside oxylipins, DHA can also be metabolised into an endocannabinoid-like derivative, docosahexaenoyl ethanolamide (DHEA), also called synaptamide. DHEA is much less well known than other *N*-acyl ethanolamides. Yet, it binds cannabinoid receptors CB1 as well as CB2 [18], promotes neurogenesis [19,20], reduces neuroinflammation [21,22], lessens sensitivity to seizures in epileptic mice [23], mitigates allergic symptoms [24], and improves glucose uptake in myoblasts [25]. Interestingly, this lipid mediator can in turn be converted into oxygenated derivatives after the action of cyclooxygenases, lipoxygenases, and cytochrome P450, suggesting a potential broader role of DHEA in tissues [26]. More generally, endocannabinoids are known for their effect on appetite and food intake, in particular 2-arachidonoyl-glycerol (2-AG) and *N*-arachidonoyl ethanolamide (AEA), also named anandamide [27,28,29]. Both are derived from arachidonic acid (ARA) and are described for their hyperphagic action as full or partial agonists of CBs [30]. Then, other compounds with cannabimimetic activities were discovered, derived from other fatty acids, including those with a *N*-acyl ethanolamide structure such as palmitoyl ethanolamide (PEA), stearoyl ethanolamide (SEA), and oleoyl ethanolamide (OEA), which are the best known [26]. Endocannabinoids and non-endocannabinoid *N*-acyl ethanolamides in particular act by activating the two receptors CB1 and CB2 [31], but also other G protein-coupled receptors such as GPR55, GPR110, and GPR119, or by stimulating ion channels (transient receptor potential vanilloid TRPV1) or even nuclear receptors (peroxisome proliferator-activated nuclear receptor PPAR-α and PPAR-γ) [26]. Endocannabinoids and related compounds thus exert various effects on cerebral functions [32,33,34,35,36], neuroinflammation [35], reproduction [37,38,39], hedonic eating behaviour [40,41], nociception and pain [42,43], immunomodulation [44], and tumour growth and angiogenesis [45,46,47]. Any inappropriate regulation of their metabolism would be associated with a pathogenic status, in particular due to a defect in their degradation leading to their unsuitable accumulation in the body.

In this study, rats received 3 g of omelette per day containing DHA in the form of crude oil or previously encapsulated with whey proteins, while a control group received a DHA-free omelette, and the profile of endocannabinoids and endocannabinoid-like compounds was analysed. This work was motivated by the effect of DHA oil encapsulation on animal food intake [16]. Indeed, the rats given the omelettes enriched with encapsulated DHA also consumed more rodent chows, while no difference was observed between the control group and the non-encapsulated DHA oil group. Since the dietary effect of a fatty acid goes beyond its own metabolism in the body, endocannabinoid derivatives have thus been quantified in order to understand the feeding behaviour of animals. In this study, the effect of DHA encapsulation in the food matrix was therefore specifically researched on the synthesis of DHEA, and more broadly of endocannabinoids of the brain, heart, and plasma.

## 2. Materials and Methods

### 2.1. Design of the Animal Experiment

**Figure 1:**
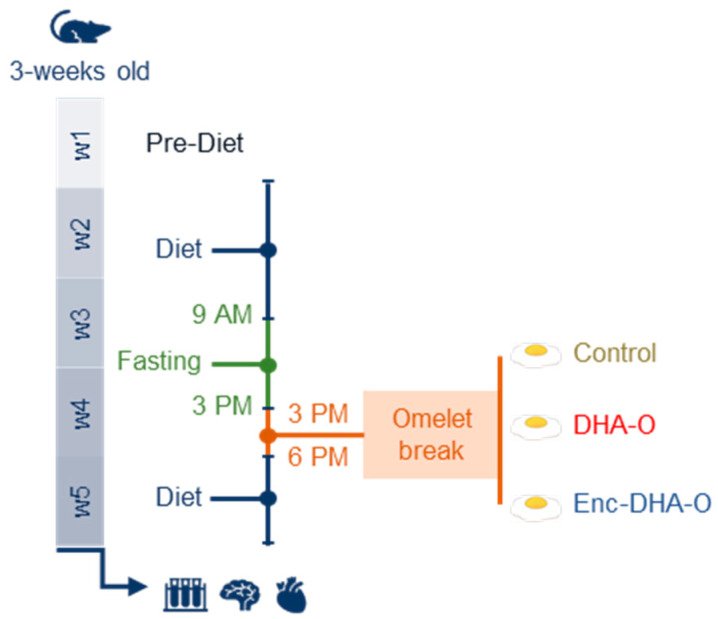
The experimental design. Rats were acclimated to the pre-diet for one week. They were then fed with the diet for four weeks. During this period, rats were daily fasted for 6 h before receiving 3 g of omelette containing DHA as crude oil (DHA-O) or as encapsulated oil (Enc-DHA-O).

Three-week-old male Wistar rats were purchased at the Janvier Labs Breeding Center (Le Genest-Saint-Isle, France). They were randomly housed in pairs into 3 groups of 8 animals. They were acclimated with a pre-diet for one week and were then fed for four weeks with the same diet but nutritionally adjusted due to the daily supply of eggs (Figure 1). 

Indeed, during this period, rats were fasted every day from 9 a.m. to 3 p.m. Then, from 3 a.m.to 6 p.m., each rat received 3 g of omelette containing either no DHA as control, non-encapsulated DHA oil (DHA-O), or encapsulated-DHA oil (Enc-DHA-O). At 6 p.m., the diet was restored until the next morning. Animals always had access to water ad libitum. Likewise, rodent chows (pre-diet and diet) were distributed ad libitum at the indicated time slots. After four weeks of the experiment, 12 h fasted rats were anaesthetised with intraperitoneal injections of ketamine (100 mg/kg, Imalgene^®^1000, Mérial, Lyon, France) and xylazine (10 mg/kg, Rompun^®^ 2%, Bayer Animal Health, Puteaux, France), and tissues were sampled as previously described [16].

### 2.2. Diets

Diets were prepared as rodent chows according to the AIN-93-G formulation and made at the Unité de Préparation des Aliments Expérimentaux of INRAE (Jouy en Josas, France) [48]. The pre-diet was composed of 20.6% proteins, 64.8% carbohydrates, and 5% lipids, whereas the diet was composed of 20.8% proteins, 65.1% carbohydrates, and 4.5% lipids. Both diets contained 5.1% fibre and 4.5% mineral and vitamin mix [16,49]. Pellets were formulated by adding a mix of vegetable oils specific to each diet (Table 1), considering the consumption of eggs (naturally rich in lipids) during the 4-week period of treatment. 

### 2.3. Fatty Acid Profile of Oils and Omelettes 

Lipids of the DHA oil (Polaris, Quimper, France) of vegetable oils used in the diets and of the omelettes were saponified with 0.5 mol/L NaOH in methanol at 70 °C for 20 min and methylated with BF3 (14% in methanol) at 70 °C for 15 min. Fatty acid methyl esters were extracted with pentane and then separated by a QP 2010-SE gas chromatograph coupled to a mass spectrometer (GC-MS, Shimadzu, Marne-La-Vallée, France) equipped with a BPX70 capillary column (120 m, 0.25 mm i.d., 0.25 µm film, SGE Trajan, Chromoptic, Paris, France) as previously described [16]. 

### 2.4. Preparation of Encapsulated DHA Oil and Omelettes 

DHA oil was daily encapsulated with heat-denatured whey protein isolate through Pickering emulsion as described previously [10]. Briefly, DHA oil was gradually added to the whey protein isolate dispersion (10 mg/mL) with a volume ratio of 0.6 to 0.4 and then mixed at 20,000 rpm for 6 min (IKA T 10 basic Ultra Turrax, IKA^®^-Werke GmbH & Co., KG., Staufen, Germany). The resulting coarse emulsion (30 mL) was homogenised in an ice bath using an ultrasonic homogeniser (Q700 Sonicator, Qsonica Sonicators, Newtown, CT, USA) with a standard 3.2 mm diameter tip half submerged. Samples were sonicated at 20 kHz with 40% amplitude for 5 min. 

The omelette was prepared in parallel with a whole egg (Moisan aviculture, Plestan, France) homogenised by Ultra Turrax (10,000 rpm, 30 s). Then, encapsulated-DHA oil (Enc-DHA-O) and heat-denatured whey protein isolate dispersion alone (Control) or completed with the DHA oil (DHA-O) were added and mixed by stirring (500 rpm, 5 min). Whey protein supplementation was therefore 300 µg per day for the three groups of animals, corresponding to less than 0.1% of egg protein. Eggs were finally moulded and cooked in a water bath (80 °C, 10 min). Thus, DHA oil droplets (1–10 µm in diameter) were surrounded by a thin layer of whey protein particles (around 42 nm in thickness) [10]. When added to the omelette, encapsulated DHA oil particles kept their spherical shape and size as previously demonstrated [17]. 

### 2.5. Endocannabinoid and Other N-Acyl Ethanolamide Analysis

Endocannabinoids and *N*-acyl ethanolamides derived from polyunsaturated fatty acids were quantified in the DHA oil, omelettes, plasma, heart, and brain by liquid chromatography combined with tandem mass spectrometry (LC-QQQ) as described elsewhere [50]. Briefly, frozen tissues (250 mg) were crushed with a FastPrep^®^-24 Instrument (MP Biomedical, Illkirch-Graffenstaden, France) in 500 µL of Hank’s balanced salt solution (Thermo Fisher Scientific, Illkirch-Graffenstaden, France). After 2 crush cycles (6.5 m/s, 30 s), cold methanol (300 µL) and internal standards (deuterium labelled compounds) were added to homogenates. Samples were then centrifuged (15 min at 2000× *g* at 4 °C) and supernatants were diluted in 2 mL water. Lipids were further separated with a solid phase extraction on an OASIS HLB 96-well plate (30 mg/well, Waters, Saint-Quentin-en-Yvelines, France) and endocannabinoids were finally eluted with 400 µL acetonitrile, 750 µL methanol, and 500 µL ethyl acetate. They were finally reconstituted in 10 µL methanol prior to LC-QQQ analysis [51]. Then, they were separated on a ZorBAX SB-C18 column (50 mm, 2.1 mm i.d., 1.8 µm film) using an Agilent 1290 Infinity HPLC system coupled to an ESI-triple quadruple G6460 mass spectrometer (Agilent Technologies, Les Ulis, France). Data were acquired in multiple reaction monitoring modes with optimised conditions (ion optics and collision energy). Peak detection, integration, and quantitative analysis were performed using Mass Hunter Quantitative analysis software (Agilent Technologies) based on calibration lines built with eicosanoid standards (Interchim, Montluçon, France). Analyses were performed at the MetaToul-Lipidomic platform (Toulouse, France). 

### 2.6. Statistics

Results are expressed as the mean ± SEM of 8 animals per group. Data analysis was performed using R software (version 3.5.2). Analysis of variance was completed by ANOVA followed by a post hoc test depending on the normality of the data distribution. The significance of the effect observed with the DHA oil or the encapsulated-DHA oil was marked by an asterisk or different letters when *p* < 0.05. The significance of the analyses of variance was indicated by stars: * when *p* < 0.05, ** when *p* < 0.01, *** when *p* < 0.001, and **** when *p* < 0.0001. “ns” means not significant. 

## 3. Results

### 3.1. Diet Characteristics

**Figure 2:**
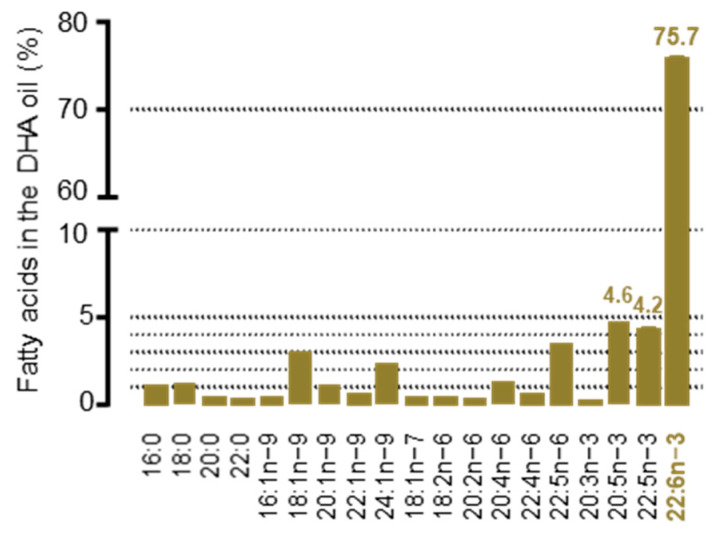
The fatty acid profile of the DHA oil. DHA oil lipids were saponified, fatty acids were methylated and then analysed by GC-MS.

First, the DHA oil used to supplement the omelettes was composed of 91% triacylglycerols and 9% diacylglycerols. It was enriched with 615 µg/mg DHA, as described in a previous work [17]. DHA represented 75.7% of the oil’s total fatty acids, with minor fatty acids, mostly omega 3, also present (Figure 2). 

Then, regarding the rodent food, animals were first acclimated for one week with the pre-diet. The composition of this diet was characterised by 17.0% linoleic acid (LA, 18:2n−6) and 3.4% α-linolenic acid (ALA, 18:3n−3) corresponding to a n−6/n−3 ratio of 5 (Table 2). This diet was conformed to the regular food for growing rats. Then, animals were fed with the diet for four weeks, the composition of which was close to the pre-diet but with a reduction in lipids considering the daily supply of eggs. The diet was thus prepared with 4.5% vegetable oil (Table 1) since eggs provided the remaining 0.5%. 

During the omelette break, rats received 3 g of eggs per day, the supplementation of which differed according to the input form of DHA oil (non-encapsulated or encapsulated). DHA was integrated into the recipe to reach 10% of the total fatty acids of the omelette, whereas the control omelette naturally contained 0.8% DHA (Table 2). Considering the diet consumption, DHA ultimately accounted for 2.5% of total fatty acids in daily dietary intake for the DHA-O and Enc-DHA-O groups, against 0.2% of total fatty acids for the control group. 

### 3.2. Dietary Intake and Animal Growth

**Figure 3:**
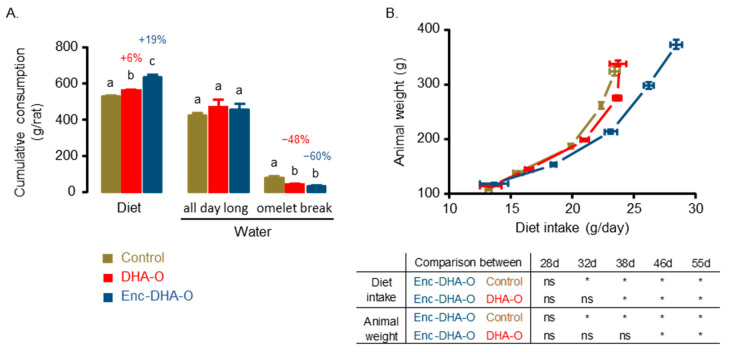
Impact of food structure on diet and water consumption (**A**) and animal weight gain (**B**). Daily diet and water consumption were monitored during the 4 weeks of treatment. The ad libitum consumption of the diet is correlated to the weight of the animals at different ages (28, 32, 38, 46, and 55 days). In (**A**), different letters between groups mean a significant difference between groups. In (**B**), the significant effect of encapsulation between the Enc-DHA-O group and the Control and DHA-O groups is indicated by an asterisk (*, *p* < 0.05). “ns” means not significant.

For four weeks, animals had access to the diet ad libitum for 15 h. They were then fasted for 6 h before receiving individually the portion of omelettes, the consumption of which was complete for each group of rats during the entire experiment. The consumption of DHA was thus on average 25 mg per day per animal. During this omelette break, animals fed with DHA consumed less water than the control group (Figure 3A). 

This contrasts with water consumption the rest of the day as no difference was observed between the groups. In parallel, food intake was significantly increased in the DHA groups, and in particular in the Enc-DHA-O group, as compared to the control group. The growth of the animals was monitored as well during the four weeks to be correlated with the diet consumption (Figure 3B). The results showed that the weight of animals in the control group increased exponentially with food consumption. This effect is linked to the growth of the animals since each point corresponds to an age. However, the growth of the control group was similar to that of the DHA-O group. On the other hand, very early the Enc-DHA-O group consumed more food, which had an impact on the weight of the animals, which was indeed greater. Since the rats were four weeks old at the start of the experiment, their growth was rapid and coincided with their weight gain. Thus, for the same diet consumption per day of 23.5 g, the animals in the control and DHA-O groups weighed more than 300 g, while those in the Enc-DHA-O group weighed almost 200 g. However, the former were 55 days old, while the latter were only 46 days old. This tends to suggest that the administration of encapsulated DHA modified the feeding behaviour of the animals. They ate more food and gained more weight because they grew faster than the other groups of animals of the same age. This is also why the average food efficacy ratio, calculated by the ratio between body weight gain and food intake, was unchanged for each diet. In view of all these results and the eating behaviour of the animals, the profile of endocannabinoids was then researched.

### 3.3. Dietary Endocannabinoids and N-Acyl Ethanolamides

DHA oil was incorporated into eggs as an oil or encapsulated. The profile of naturally occurring endocannabinoids and *N*-acyl ethanolamides was first checked in DHA oil and then in omelettes to distinguish the contribution of oil and that of egg (Table 3). 

Five endocannabinoids and other *N*-acyl ethanolamides were found in the DHA oil, including one derived from DHA, called DHEA, representing only 0.2% of total endocannabinoid derivatives. The major compound was 2-AG, accounting for 98.7% of the total. The others were SEA, OEA, and dihomo-γ-linolenoyl ethanolamide (DLE), representing 1.2%. DHA oil contained 9.95 ng endocannabinoids per mg. The plain omelette contained endocannabinoids and *N*-acyl ethanolamides as well but with a bigger diversity of species, AEA and EPEA being absent in the DHA oil and only provided by the omelette. The major compound was also 2-AG (77.2%), and then SEA (10.3%) and OEA (8.7%). 

When omelettes were supplemented with DHA oil, the proportion of 2-AG increased from 77.2% to around 83%. DHA-derived DHEA only accounted for 0.5–0.6% of total endocannabinoid derivatives. Finally, daily omelette consumption provided 1.5 µg of endocannabinoids and *N*-acyl ethanolamides in the control group versus 1.8 µg in the DHA-O and Enc-DHA-O groups. On this base, the profiles of endocannabinoids and *N*-acyl ethanolamides of animals were further determined after four weeks of diet with omelettes.

### 3.4. Effect of Diets on Tissue Endocannabinoid and N-Acyl Ethanolamide Profiles of Rats

Endocannabinoids and *N*-acyl ethanolamides were quantified in the brain, heart and plasma of animals. Firstly, the main endocannabinoid found in tissues was 2-AG, which was already the most abundant one in the omelette serving (Table 4). The other *N*-acyl ethanolamides were identical between omelettes and tissues, with the exception of DLE and PEA. Indeed, DLE was present in the omelettes but not in the tissue, and conversely, PEA was absent from the omelettes but found in the tissues. Secondly, the concentration and profile of endocannabinoids and *N*-acyl ethanolamides were differently affected by omelette diets in different tissues. Endocannabinoid and *N*-acyl ethanolamide concentration decreased by approximately 50% in plasma and brain when omelettes were fortified with DHA, with or without DHA oil encapsulation. No such effect was observed in the heart. 

In the brain, 2-AG represented 82% of the total endocannabinoids and *N*-acyl ethanolamides in the control group, DHEA 3%, and the others 15%. When animals were treated with the omelette containing DHA, concentrations of SEA, OEA, 2-AG and AEA were significantly decreased by 48%, 43%, 60%, and 37%, respectively, between the control group and the DHA-O group, and a little more with the DHA oil encapsulation, while the PEA remained unchanged (Table 4 and Supplemental Figure S1A). Surprisingly, the concentration of DHEA was also significantly decreased by 38% between control and DHA-O, but without significant effect of DHA oil encapsulation. 

In the plasma, 2-AG represented 64% of the total endocannabinoids and *N*-acyl ethanolamides, OEA 26%, but DHEA was absent. When animals were treated with an omelette containing DHA, two major effects were observed (Table 4 and Supplemental Figure S1C). The first effect was the significant decrease in 2-AG between the control group and the DHA groups (−77% and −72% in the DHA-O and Enc-DHA-O groups, respectively). The second effect was the appearance of DHEA in the DHA groups, representing 2.3% of the total in plasma, with no difference between encapsulated or unencapsulated DHA oil. 

Finally, in the heart composed of 87% of 2-AG, 1.6% of DHEA, and 12% of the other endocannabinoids and *N*-acyl ethanolamides, the effect of the diets contrasted with the two previous tissues. A bell-shaped effect was observed between the three groups (Table 4 and Supplemental Figure S1B). For most of the endocannabinoids and *N*-acyl ethanolamides, the concentrations were similar between the control group and the Enc-DHA-O group, while a 50% decrease was observed in the DHA-O group. However, one exception concerned DHEA as the concentration of DHEA was the same in the control and DHA-O groups, but 2.4-fold more important when DHA oil was encapsulated.

**Figure 4:**
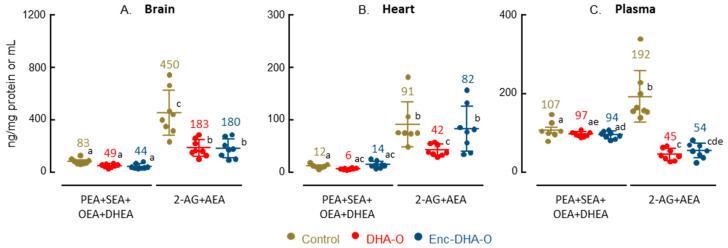
Endocannabinoids and *N*-acyl ethanolamides in brain (**A**), heart (**B**), and plasma (**C**) of animals after the 4 weeks of treatment. Fatty acid derivatives are grouped by family, endocannabinoids and *N*-acyl ethanolamides. Levels are expressed in ng/mg of protein for brain and heart, and in ng/mL for plasma. The average is indicated by a number for each group of animals. Different letters between groups mean a significant difference between groups.

Globally, organs responded differently to different omelettes. The overall profile of *N*-acyl ethanolamides was not significantly modified according to the types of omelettes, unlike that of endocannabinoids (Figure 4). Overall, DHA supplementation reduced tissue levels of endocannabinoids, with the exception of the hearts of animals supplemented with encapsulated DHA. Although the dispersion of the values was important, the cardiac endocannabinoid contents were similar between the control and the Enc-DHA-O groups. 

**Figure 5:**
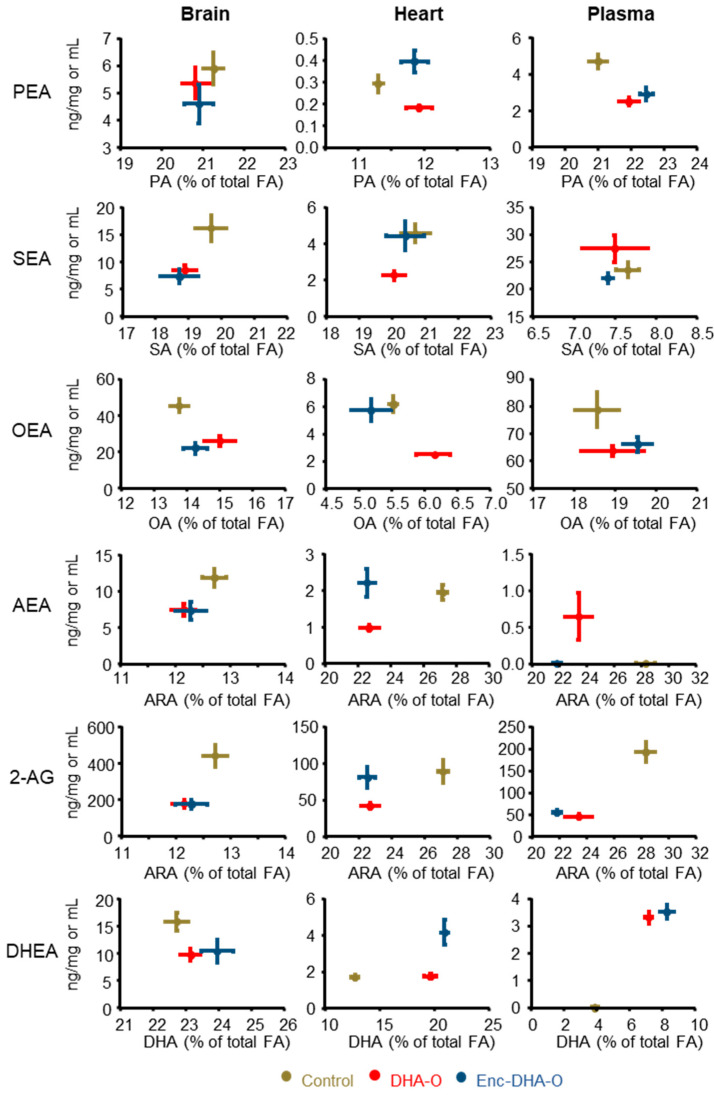
Endocannabinoids and *N*-acyl ethanolamides correlated to the respective precursor fatty acids. The content of endocannabinoids and *N*-acyl ethanolamides is expressed as a function of the proportion of the precursor fatty acid previously quantified [16].

Then, to understand such effects, the endocannabinoid and *N*-acyl ethanolamide concentrations were compared to the fatty acid profile [16]. We found that the drastic reduction in endocannabinoid derivatives with the DHA-enriched diet in the brain was independent of their fatty acid precursors (Figure 5 and Supplemental Figure S1). 

On the contrary, in plasma, 2-AG and DHEA were more prominent when the concentrations of ARA and DHA, respectively, were higher. The case of the heart was different again, since several situations were observed. The DHA-O group was particularly isolated from the other two in terms of endocannabinoid and *N*-acyl ethanolamide concentrations since they were lower in this group, yet with the same proportion of precursor fatty acid as in the other two groups (PA, SA, OA) or in the Enc-DHA-O group (AEA, 2-AG). The exception of DHEA was again present because the encapsulation of DHA oil showed a higher concentration of DHEA with, however, a proportion of DHA similar to that of the unencapsulated DHA group.

The comparisons consider the percentages of fatty acids in the studied organs, but the effect is observed in a similar way when the results are expressed in fatty acid concentrations (not shown here). Thus, all of these results showed that the diet enriched with DHA could modulate the tissue concentrations of endocannabinoids or *N*-acyl ethanolamides, but that the profile of precursor fatty acids was not in itself a predictive factor of these concentrations of endocannabinoids or *N*-acyl ethanolamides. Thus, a stable fatty acid profile can hide very contrasting profiles in metabolic derivatives.

## 4. Discussion

The purpose of this study was to show the impact of the delivery of DHA through the ingestion of fortified omelettes on its metabolism, more specifically on the endocannabinoid system. First, the analysis revealed the dietary presence of endocannabinoids and other *N*-acyl ethanolamides in the eggs and the DHA oil used for the experiment. This oil is thus constituted for a large part of 2-AG but devoid of AEA, and has a low content of *N*-acyl ethanolamides, among which is DHEA derived from DHA. The egg, on the other hand, is richer with a more diversified profile in endocannabinoids and *N*-acyl ethanolamides. To our knowledge, there has never been a report on the presence of such lipid mediators in foods before. The endocannabinoids and *N*-acyl ethanolamides analysed represent only 0.00065% of the total fatty acids. This proportion, 20–25% higher with DHA oil in omelettes, is very low but exists, and it remains not even considered during nutritional trials, in particular with fish oils or oils rich in DHA.

The presence of these lipid mediators may contribute to some of the effects observed after supplementation with these oils rich in omega 3 fatty acids. There are no data on the stability and the digestion and absorption process of dietary endocannabinoids and *N*-acyl ethanolamides. Therefore, the impact of food intake cannot be predicted. In contrast, the endocannabinoid system is well described in the gastrointestinal tract, notably in the regulation of food intake and energy balance. DiPatrizio and co-authors have in particular demonstrated the importance of the regulatory control of the endocannabinoid system on dietary lipid intake [52]. They showed that oral exposure to dietary oil stimulates the mobilisation of 2-AG and AEA in the small intestine in rats. They described the endocannabinoid system in the gut as a key component of the positive feedback mechanism that drives fat intake. Moreover, both CB1 and CB2 are present in the submandibular gland of rats, and when activated by 2-AG and AEA, they inhibit salivary secretion [53]. Salivation varies also according to the smells of food, which also conditions the appetite [54]. We can therefore conclude that in this study, the animals had a slightly greater diet intake with the DHA oil-enriched omelettes because these were richer in lipids and contained approximately 20% more endocannabinoids and *N*-acyl ethanolamides, a difference which may have impacted over time the oro-sensory properties of the following meals. However, this does not explain the specific effect observed with DHA encapsulation compared to the control group. Encapsulation protects the DHA oil in the mouth since the whey proteins used for encapsulation are not digested during the oral phase, so the observed effect on food intake cannot be limited to the oral process during digestion.

Our previous results showed that encapsulation leads to a greater release of DHA during digestion. DHA itself activates the expression of cannabinoid receptors such as CB1 [55]. The greater release of DHA in the case of encapsulation of DHA oil would thus induce a greater increase in the number of CB1 receptors in the gastrointestinal tract, all the more favouring the stimulation of the reward system. It is also conceivable that the early release of endocannabinoids and *N*-acyl ethanolamides from the omelette with encapsulated DHA oil further promotes the food intake after the omelette break. Endocannabinoids are indeed known to stimulate the secretion of the orexigenic hormonal peptide ghrelin by activating CB1 [56]. This effect, although pre-prandial, would be accentuated by the faster and greater release of the constituents of the encapsulated DHA oil, and this is all the more so in fasting conditions where ghrelin is strongly secreted. The signal could be temporarily maintained in the case of encapsulated DHA oil, leading to a different feeding behaviour afterwards. Thus, the animals fed with the omelette enriched with encapsulated DHA oil would have more desire to eat following a stronger activation of the reward system consecutive to a greater activation of CB1 in the gastrointestinal tract, which they did by consuming more food on a daily basis. 

To confirm this hypothesis, it would have been interesting to measure food intake more precisely, to see if this difference between the groups was present only after the omelette break or continued throughout the day. In addition, ghrelin would also be involved in the growth process since it would activate the growth hormone release [57]. This would explain as well the increased growth of animals subjected to omelettes with encapsulated DHA oil. However, explaining the precise mechanisms requires further studies. We did not find any literature on the effect of DHA on the growth and body development of offspring. No accurate effect of DHA on food intake and eating behaviour has been described either. On the other hand, numerous studies report the effect of DHA on weight loss. DHA supplementation actually induces weight loss or helps reduce weight gain. However, the effects are generally observed in obese or overweight adult models. Sometimes, even supplements are based on fish oils and not purified DHA oils [58,59,60]. In any case, these observations do not go in the direction that we observed in our experimental conditions. Notwithstanding, it remains important to consider the study model. Irving et al. have for example shown that DHA and EPA administered as food supplements promoted weight gain and tended to improve appetite in patients with Alzheimer’s disease [61]. Likewise, Pauter et al. studied Elovl2^-/-^ mice deficient in Elovl2 elongase involved in DHA synthesis [62,63]. When administering dietary DHA, tissue DHA levels are recovered, but the animals also gain weight. The authors demonstrated that DHA potentiates weight gain when animals are subjected to a high-sucrose diet or a high-fat diet, and concluded that DHA exhibits a positive effect on lipid accumulation in Elovl2-deficient mice. Nonetheless, this concerns young adult mice which are no longer growing. In our experimental conditions, it seems that the greater release of DHA with encapsulation during intestinal digestion potentiated a similar action through a multifactorial mechanism, independently of its metabolism, but first at the peripheral level by possibly modulating the gastrointestinal secretion of satiation or appetite hormones. Gut-brain endocannabinoid signalling indeed controls the consumption of palatable foods. This signalling is strongly associated with pathways regulating food intake and the satiety cascade [64]. The effect of DHA appears to fit into this complex signalling due to the expression of CB1 receptors in the gastrointestinal tract. Thus, hormone peptides whose release is induced by nutrients in the intestinal epithelium modulate the activation of their vagal afferent neuron receptors, or endocannabinoids bind directly to cannabinoid receptors expressed on vagal afferent neurons [65]. On this basis, it is therefore conceivable that the encapsulated DHA oil, rich in DHA but also in endocannabinoids, participated in one of these peripheral mechanisms, ultimately promoting greater food intake by the animals. 

The other important factor in this study is the fast period before the omelette administration. The purpose of this daily fast was to separate the digestion of the omelettes from that of the diet. Indeed, the delivery of DHA via food aims to modify the bioaccessibility of DHA and therefore the digestion process, thus making it possible to modulate the bioavailability of DHA and its subsequent metabolism. If this objective was achieved by avoiding any bias linked to omelette digestion coupled with that of diet, fasting probably led to synergistic effects between differentially digested DHA oil and physiological effects following fasting. To our knowledge, only one study highlights the effects of DHA in a fasting context. Deval and co-authors showed this link with young mice fed a DHA-enriched diet for 8 weeks and then fasted for 48 h [66]. They thus showed that a food pre-treatment in DHA before fasting allowed a more efficient mobilisation of energy reserves, thus preserving muscle mass from proteolysis. In our study, optimal growth conditions seem to overlap: growth rodent food, DHA supplementation, and daily partial fasting. This condition of repeated fasting followed by consumption of palatable food (omelette) would reinforce the activation of the endocannabinoid system, particularly in the case of the encapsulation of DHA oil given its greater digestion. Indeed, Gomez and co-authors showed that levels of 2-AG and AEA in the small intestine increase during food deprivation and decrease upon refeeding [67]. Here, the effect on the endocannabinoidome would be prolonged with the digestive release of DHA after the period of partial fasting, particularly with the encapsulation of DHA. Now, although the benefits of fasting are multiple, the effect of fasting on growth is not described in the literature. Therefore, studying the impact of a nutrient such as DHA in a fasting condition will require other investigations to understand the molecular mechanisms. Fasting conditions could in particular be re-evaluated in considering the circadian cycle and the twilight and nocturnal activity of the rat.

In this study, DHA supplementation reduced water consumption during the omelette break. This effect only really takes place after 15 days of dieting and does not affect daily water consumption. The activation of CB1 participates in the control of water consumption, but this effect depends on the cell type and the brain area overexpressing CB1 [68]. Animals subjected to DHA-enriched omelettes showed markedly reduced levels of endocannabinoids in the brain. This reduction could explain the decrease in the animals’ water consumption. However, this difference only occurs during the omelette break, which also underlies a link with the consumption of DHA-enriched omelettes. The effect would therefore be rather peripheral and not only central, possibly mediated by a difference in digestion between the DHA oil and encapsulated DHA oil. Again, a difference in activation of ghrelin secretion could be involved in the observed effect since the hormonal peptide is also known to inhibit water consumption under the study conditions [69]. 

Under our study conditions, DHA supplementation modified the endocannabinoid and *N*-acyl ethanolamide profile in the three tissues analysed. Overall, DHA induces a drastic reduction in these fatty acid derivatives in the brain and plasma, the profile of the heart being slightly different. This effect mainly concerns derivatives of ARA. The literature shows that DHA supplementation alters the profile of cannabinoid derivatives, but not always so drastically and also depending on the cerebral area [70,71,72]. In addition, DHA supplementation induces an increase in DHEA in the plasma and the heart, but not in the brain where a reduction is on the contrary observed. Again, this contrasts with the literature where the concentration of DHEA is increased even in the brain after dietary DHA supplementation. Mock and co-authors also point out that the cerebral concentration ratio between DHEA and AEA is generally between 2 and 10 in mice, unlike in plasma [26]. This ratio is not observed in this study where, moreover, neither DHEA nor AEA were quantified in the control plasma. This is also not the case in other studies on the rat model, which show that the concentrations of endocannabinoids and *N*-acyl ethanolamides vary according to the brain areas studied, with ratios that can even be very different [73,74]. Now concerning the encapsulation of DHA oil, only the heart has been impacted with a very specific profile. The reduction in endocannabinoids and *N*-acyl ethanolamides with DHA supplementation was not observed when the DHA oil was encapsulated, to the point of returning to a control level. Such results remain very difficult to interpret. It can however be noted that the DHEA content in the heart is specifically and greatly enriched in the case of encapsulation of DHA oil. To our knowledge, there are no data concerning the effect of DHEA on cardiac function. The activation of CB1 and CB2 has been further studied in the presence of AEA and 2-AG. Overall, the works show that the endocannabinoid system distinctly involves CB1 and CB2, but is differently engaged depending on heart disease and cardiac function [75]. In a healthy heart, the activation of CB1/CB2 would not participate in the electrophysiological process nor in the regulation of the cardiac rhythm. On the other hand, in cardiac disorders or after a myocardial infarction, circulating levels of endocannabinoids would be increased, which would improve cardiac performance. Thus, in the event of a cardiac dysfunction, inhibiting CB1 would be cardioprotective against ischemia-reperfusion injury, heart failure, arrhythmia, or cardiomyopathies, while activating CB2 would be cardioprotective. The regulation of cardiac contractility by the endocannabinoid system nevertheless remains complex because it would involve the central nervous system but also cardiac mechanisms that are still poorly described. However, we can remember that DHEA improves the entry of glucose into the myoblasts [25] and assume that the increase in DHEA in the heart muscle is in favour of an improvement in cardiac contractions when the animals consume the encapsulated DHA. In all cases, administering an encapsulated DHA oil under our experimental conditions revealed physiological effects which will require further studies to understand the specific mechanisms of action.

## 5. Conclusions

Intake of encapsulated DHA oil resulted in increased growth of growing rats associated with increased food consumption. The levels of endocannabinoids and *N*-acyl ethanolamides derived from omega 6 fatty acids were strongly reduced in plasma, heart, and brain with DHA supplementation, but without great impact of encapsulation. The effect observed would therefore be inherent in the effect of the structure of the food during digestion. Thus, a higher concentration of DHA and cannabinoid derivatives released during digestion thanks to the encapsulation of DHA oil would increase the gastrointestinal stimulation of the cannabinoid system. This effect would be all the more accentuated when the animals undergo a partial fast preceding the omelette break. The various data obtained suggest that the observed physiological effects were the result of the synergistic action of the nutritional conditions of animal growth, daily partial fasting, and the differential digestion of DHA oil. Monitoring the hormones that regulate food intake during periods of fasting and omelette breaks would provide a better understanding of the molecular mechanisms involved in animal development.

## Figures and Tables

**Table 1:** The composition of diets and oil mix. The composition of dry diets based on the AIN-93-G is presented for the pre-diet and the diet. The diet contained a mixture of vegetable oils formulated according to the daily consumption of eggs.

	%	Pre-Diet	Diet
Diet mix	Starch	54.6	54.9
	Sucrose	10.2	10.2
	Cellulose	5.1	5.1
	Casein	20.6	20.8
	Oil mix	5.0	4.5
	Mineral mix	3.5	3.5
	Vitamin mix	1.0	1.0
Oil mix	Linseed	1.4	2.9
	Sunflower	8.6	8.8
	Olive	13.5	12.6
	Rapeseed	25.0	25.2
	Palm	51.5	50.5

**Table 2:** The fatty acid composition of the diets and the omelettes. Lipids from rodent chows and omelettes were extracted. The fatty acid (FA) profile was then determined by GC-MS for the acclimation diets, then for the experiment diet, as well as for the omelettes provided during the omelet break. For the diet with omelette consumption, the fatty acid profile was further calculated based on the diet consumption per group and the daily 3 g omelette.

	Diets		Omelettes			Diet with Omelettes		
FA (%)	Pre-diet	Diet	Control	DHA-O	Enc-DHA-O	Control	DHA-O	Enc-DHA-O
saturates	31.0	30.5	33.6	29.9	29.6	31.2	30.9	30.9
n−7	1.6	1.6	4.9	4.4	4.4	2.4	2.3	2.3
n−9	47.0	46.4	43.0	38.9	38.3	45.6	45.7	45.7
n−6	17.0	17.2	17.1	15.6	15.5	17.2	17.3	17.2
18:3n−3	3.4	4.3	0.5	0.4	0.5	3.4	3.4	3.5
20:5n−3	0.0	0.0	0.0	0.5	0.5	0.0	0.1	0.1
22:5n−3	0.0	0.0	0.0	0.5	0.5	0.0	0.1	0.1
22:6n−3	0.0	0.0	0.8	9.8	10.6	0.2	2.5	2.5
n−3	3.4	4.3	1.3	11.2	12.1	3.6	6.1	6.2

**Table 3:** Content of endocannabinoids and *N*-acyl ethanolamide naturally present in DHA oil and in prepared omelettes. Endocannabinoids (EC) and *N*-acyl ethanolamides (NAE) derived from precursor fatty acids were extracted from the DHA oil and the different omelettes to be assayed by LC-QQQ. The concentrations are presented in pg per mg of food or in ng per daily portion since each animal received every day 3 g of omelette for 4 weeks.

Concentrations of endocannabinoids and *N*-acyl ethanolamides in pg/mg				
		**omelette**		
EC + NAE	**DHA oil**	Control	DHA-O	Enc-DHA-O
PEA (from PA)	0.0	0.0	0.0	0.0
SEA (from SA)	56.7	50.6	43.3	49.4
OEA (from OA)	48.0	42.6	37.0	43.3
DLE (from DGL)	10.0	8.6	6.2	5.3
AEA (from ARA)	0.0	4.1	4.9	6.5
2-AG (from ARA)	9817.8	378.4	515.5	474.7
EPEA (from EPA)	0.0	3.1	2.0	2.5
DHEA (from DHA)	16.2	2.6	2.9	3.5
Sum (ng/mg)	9.95	0.49	0.61	0.59
Concentrations of endocannabinoids and *N*-acyl ethanolamides in ng/serving				
	**expected from** **DHA oil**	**omelette**		
EC + NAE		Control	DHA-O	Enc-DHA-O
PEA (from PA)	0.0	0.0	0.0	0.0
SEA (from SA)	2.7	151.7	129.8	148.1
OEA (from OA)	2.2	127.9	111.1	129.9
DLE (from DGL)	0.5	25.9	18.5	15.9
AEA (from ARA)	0.0	12.4	14.6	19.6
2-AG (from ARA)	459.7	1135.3	1546.6	1424.1
EPEA (from EPA)	0.0	9.2	6.1	7.6
DHEA (from DHA)	0.8	7.9	8.8	10.5
Sum (µg/serving)		1.47	1.84	1.76

**Table 4:** The endocannabinoid and *N*-acyl ethanolamide profile of rat tissues. Endocannabinoids (EC) and *N*-acyl ethanolamides (NAE) derived from precursor fatty acids (FA) were extracted from tissues after the 4 weeks of treatment to be assayed by LC-QQQ. The concentrations are presented according to the tissues and the type of omelette. Results are expressed in ng per mg of protein for the brain and the heart, and in ng per mL of plasma. Different letters between groups mean a significant difference between groups. The stars show that the analyses of variance are significant with *p* < 0.05 (*), *p* < 0.01 (**), *p* < 0.001 (***), *p* < 0.0001 (****). “ns” means not significant.

**Brain**													
EC + NAE	**Control**				**DHA-O**				**Enc-DHA-O**				Anova
PEA (from PA)	5.88	±	0.58		5.34	±	0.56		4.58	±	0.72		ns
SEA(from SA)	16.04	±	2.39	a	8.35	±	1.00	b	7.34	±	1.30	b	**
OEA (from OA)	44.96	±	3.59	a	25.70	±	2.83	b	21.70	±	2.97	b	****
DLE (from DGL)	0.00	±	0.00	a	0.04	±	0.02	b	0.00	±	0.00	a	**
AEA (from ARA)	11.81	±	1.25	a	7.41	±	0.81	b	7.26	±	1.28	b	*
2-AG (from ARA)	438.56	±	60.21	a	175.63	±	22.85	b	172.59	±	24.93	b	***
EPEA(from EPA)	0.00	±	0.00		0.00	±	0.00		0.00	±	0.00		-
DHEA (from DHA)	15.73	±	1.67	a	9.67	±	1.04	b	10.31	±	2.02	a*b	*
Sum	532.98	±	66.46	a	232.14	±	23.90	b	223.79	±	31.55	b	****
										* *p* = 0.069			
**Heart**													
EC + NAE	**Control**				**DHA-O**				**Enc-DHA-O**				Anova
PEA (from PA)	0.29	±	0.04	ab	0.18	±	0.01	a	0.39	±	0.05	b	*
SEA(from SA)	4.14	±	0.63		2.15	±	0.24		4.27	±	0.75		ns
OEA (from OA)	5.68	±	0.74	a	2.44	±	0.14	b	5.54	±	0.77	a	**
DLE (from DGL)	0.00	±	0.00		0.00	±	0.00		0.00	±	0.00		-
AEA (from ARA)	1.94	±	0.22	ab	0.97	±	0.09	a	2.20	±	0.38	b	*
2-AG (from ARA)	88.64	±	16.20		41.33	±	3.81		80.31	±	14.99		ns
EPEA(from EPA)	0.00	±	0.00		0.00	±	0.00		0.00	±	0.00		-
DHEA (from DHA)	1.67	±	0.18	a	1.72	±	0.13	a	4.12	±	0.69	b	*
Sum	102.36	±	17.76		48.79	±	4.26		96.83	±	17.53		ns
**Plasma**													
EC + NAE	**Control**				**DHA-O**				**Enc-DHA-O**				Anova
PEA (from PA)	4.67	±	1.04	a	2.50	±	0.21	b	2.89	±	0.35	b	***
SEA(from SA)	23.45	±	3.72		27.37	±	2.45		21.92	±	0.92		ns
OEA (from OA)	78.46	±	17.41	a	63.54	±	1.73	b	65.95	±	2.81	b	*
DLE (from DGL)	0.00	±	0.00		0.02	±	0.02		0.00	±	0.00		-
AEA (from ARA)	0.00	±	0.00	a	0.64	±	0.32	a*	0.00	±	0.00	a	***
2-AG (from ARA)	191.86	±	66.03	a	44.14	±	5.54	b	53.85	±	6.59	b	***
EPEA(from EPA)	0.00	±	0.00		0.00	±	0.00		0.00	±	0.00		-
DHEA (from DHA)	0.00	±	0.00	a	3.29	±	0.22	b	3.48	±	0.25	b	***
Sum	298.44	±	70.37	a	141.49	±	6.03	b	148.10	±	5.16	b	***
								* *p* = 0.055					

## Data Availability

Not applicable.

## Supplementary Materials

The following supporting information can be downloaded at: https://www.mdpi.com/article/10.3390/nu15214622/s1, Supplemental Figure S1. Principal component analysis of lipids and rats was determined based on the type of omelette for each tissue.

## Abbreviations

AEA: arachidonoyl ethanolamide; 2-AG: 2-arachidonoyl-*sn*-glycerol; ARA: arachidonic acid (20:4n−6); CB: cannabinoid receptor; DGL: dihomo-γ-linolenic acid (20:3n−6); DHA: docosahexaenoic acid (22:6n−3); DHA-O: omelette containing crude DHA oil; DHEA: docosahexaenoyl ethanolamide; DLE: dihomo-γ-linolenoyl ethanolamide; EC: endocannabinoid; Enc-DHA-O: omelette containing encapsulated DHA oil; EPA: eicosapentaenoic acid; EPEA: eicosapentaenoyl ethanolamide; FA: fatty acid; GPR: G protein-coupled receptor; GC-MS: gas chromatography coupled to mass spectrometry; LC-QQQ: liquid chromatography combined with tandem mass; NAE: *N*-acyl ethanolamide; OA: octadecenoic acid (18:1n−9); OEA: octadecenoyl ethanolamide; PA: palmitic acid (16:0); PEA: palmitoyl ethanolamide; PPAR: peroxisome proliferator-activated nuclear receptor; SA: stearic acid (18:0); SEA: stearoyl ethanolamide; TRPV: transient receptor potential vanilloid.
