# Supplementary material for: Effect of Docosahexaenoic Acid Encapsulation with Whey Proteins on Rat Growth and Tissue Endocannabinoid Profile
Source: Nutrients. 2023 Oct 31;15(21):4622. doi: 10.3390/nu15214622 (PMC10650154; doi:10.3390/nu15214622)
Supplement: Supplementary file 1 [file nutrients-15-04622-s001.zip › nutrients-2659620-supplementary.pdf]

Figure S1

Principal component analysis of lipids and rats was determined based on the type of omelette for each tissue.

In the brain (**A**), DHA is not particularly well represented nor is it particularly correlated with DHEA or other cannabinoid derivatives. On the other hand, it is negatively correlated with ARA. Concerning the individuals, the control group is distinct from the two other groups supplemented with DHA.

In the heart (**B**), DHA is no more correlated with DHEA or even with other cannabinoid derivatives. On the other hand, the three groups of individuals are distinguished by the analysis of endocannabinoids and N-acyl ethanolamides with their precursor fatty acids.

Finally, in plasma (**C**), DHA is positively correlated with DHEA but negatively with 2-AG, OEA and PEA. The groups supplemented with DHA are distinct from the control, but without impact of the encapsulation on the distribution of the individuals.

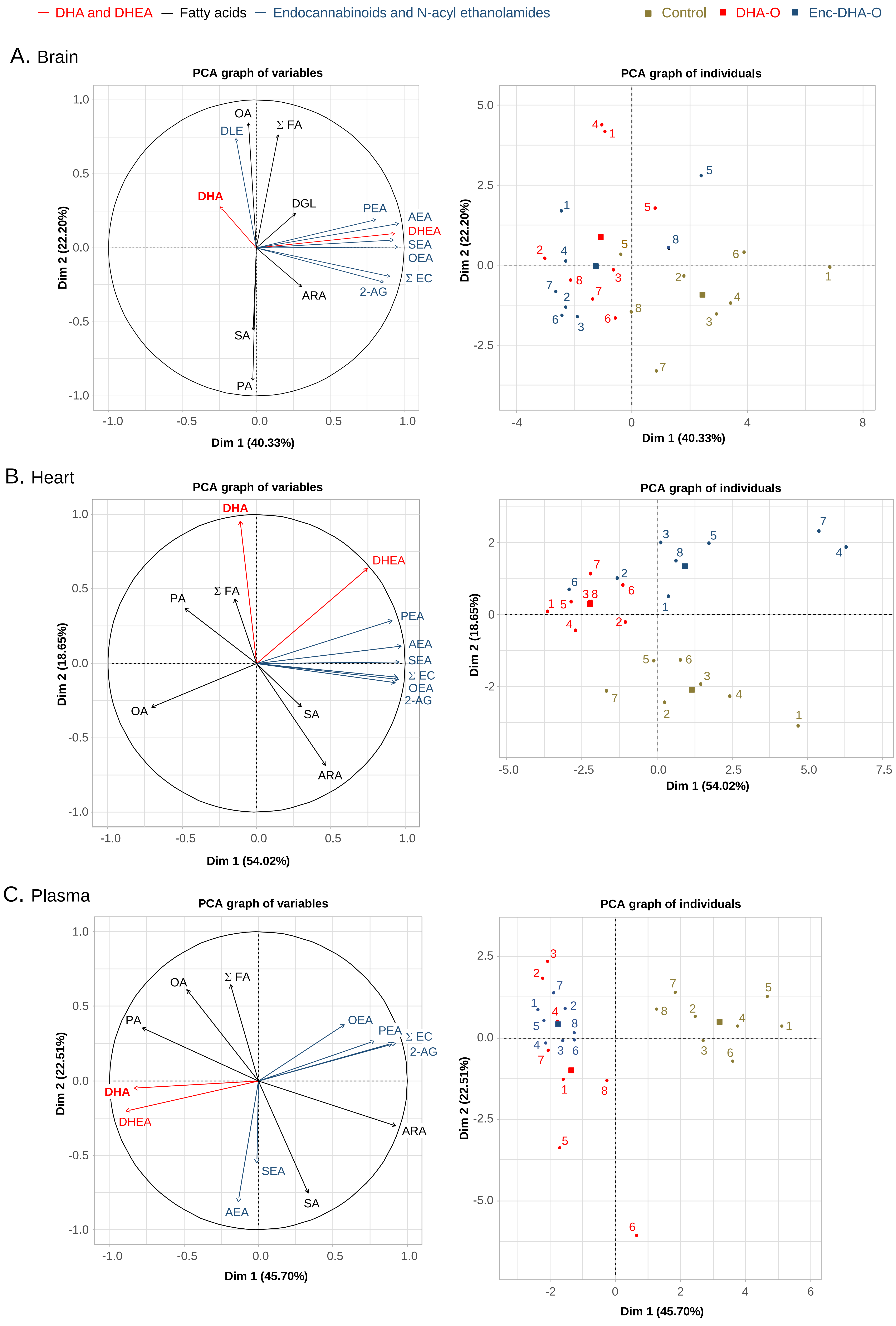

— DHA and DHEA — Fatty acids — Endocannabinoids and N-acyl ethanolamides
Control DHA-O Enc-DHA-O
A. Brain
PCA graph of variables
OA
Σ FA
DLE
DHA
DGL
PEA
AEA
DHEA
SEA
OEA
Σ EC
2-AG
ARA
SA
PA
Dim 1 (40.33%)
Dim 2 (22.20%)
PCA graph of individuals
Dim 1 (40.33%)
Dim 2 (22.20%)
B. Heart
PCA graph of variables
DHA
DHEA
PA
Σ FA
PEA
AEA
SEA
Σ EC
OEA
2-AG
OA
SA
ARA
Dim 1 (54.02%)
Dim 2 (18.65%)
PCA graph of individuals
Dim 1 (54.02%)
Dim 2 (18.65%)
C. Plasma
PCA graph of variables
OA
Σ FA
PA
OEA
PEA
Σ EC
2-AG
DHA
DHEA
ARA
SEA
AEA
SA
Dim 1 (45.70%)
Dim 2 (22.51%)
PCA graph of individuals
Dim 1 (45.70%)
Dim 2 (22.51%)
